# *At5g63290* does not encode coproporphyrinogen III oxidase

**DOI:** 10.1093/plphys/kiaf046

**Published:** 2025-01-29

**Authors:** Wenjuan Ji, Huijuan Wang, Ning An, Xuan Zhou, Bingxiao Wen, Bernhard Grimm, Zhenhua Liu

**Affiliations:** Joint Center for Single Cell Biology, Shanghai Collaborative Innovation Center of Agri-Seeds, School of Agriculture and Biology, Shanghai Jiao Tong University, Shanghai 200240, China; Joint Center for Single Cell Biology, Shanghai Collaborative Innovation Center of Agri-Seeds, School of Agriculture and Biology, Shanghai Jiao Tong University, Shanghai 200240, China; Joint Center for Single Cell Biology, Shanghai Collaborative Innovation Center of Agri-Seeds, School of Agriculture and Biology, Shanghai Jiao Tong University, Shanghai 200240, China; Joint Center for Single Cell Biology, Shanghai Collaborative Innovation Center of Agri-Seeds, School of Agriculture and Biology, Shanghai Jiao Tong University, Shanghai 200240, China; Institute of Biology/Plant Physiology, Humboldt Universität zu Berlin, 10099 Berlin, Germany; Institute of Biology/Plant Physiology, Humboldt Universität zu Berlin, 10099 Berlin, Germany; Joint Center for Single Cell Biology, Shanghai Collaborative Innovation Center of Agri-Seeds, School of Agriculture and Biology, Shanghai Jiao Tong University, Shanghai 200240, China

## Abstract

At5g63290 is not responsible for the decarboxylation of coproporphyrinogen III, prompting a revision of the tetrapyrrole biosynthesis pathway in *Arabidopsis thaliana*.

Dear Editor,

Tetrapyrrole synthesis plays essential roles in the primary metabolism across all life kingdoms. The main end products of the biosynthetic pathway are chlorophyll and heme, but photosynthetic organisms also use several other tetrapyrroles, such as siroheme and phytochromobilin (Belot et al. 2024; Gao et al. 2024). It has been demonstrated that the synthesis of chlorophyll and heme includes the oxidative decarboxylation of coproporphyrinogen III to produce protoporphyrinogen IX (Fig. 1A). While an oxygen-dependent coproporphyrinogen III oxidase (also named HemF) has been characterized in several bacteria and across Eukaryotes, including thale cress (*Arabidopsis thaliana*) (Ishikawa et al. 2001), mice (*Rattus norvegicus*) (Mori et al. 2013), yeast (*Saccharomyces cerevisiae*) (Phillips et al. 2004), and *Escherichia coli* (Breckau et al. 2003), the oxygen-independent coproporphyrinogen III oxidase (also named HemN) has been thoroughly identified only in *E. coli*, as demonstrated by others and our own work (Fig. 1A) (Layer et al. 2002; Ji et al. 2020). The HemN requires *S*-adenosyl-l-methionine (SAM), a reductant, and additional cytoplasmatic components for catalysis and carries/contains a Fe–S cluster (Layer et al. 2002). Whether an oxygen-independent CPO exists in Eukaryotes, however, has not been clearly established. Recently, Pratibha et al. (2017) identified a potential HemN-like enzyme (At5g63290) from the model plant Arabidopsis. In their findings, the homozygous mutant line, designated *Athemn1*, was lethal, and the heterozygous progenies of the mutant showed defects in gametophyte development (Fig. 1B). However, when analyzing the same T-DNA insertion line used by Pratibha et al. and in addition to 3 independent mutant lines generated with CRISPR/Cas9, no obvious growth and developmental phenotypes were detectable for the HemN-deficient mutant lines (Fig. 1, C to E; Supplementary material and Methods). By analyzing the phylogeny of HemN-like proteins across all kingdoms (Cheng et al. 2022), our results indicate that At5g63290 is not grouped with HemN, but rather with the HemW clade (Fig. 2A; Supplementary material and Methods). Initial biochemical analysis of *Lactococcus lactis* revealed that HemW is a heme chaperone in heme transfer (Haskamp et al. 2018). We further purified *E. coli* HemN and At5g63290 and performed in vitro enzyme assays. While we clearly observed functional CPO activity of *E. coli* HemN, we failed to detect decarboxylation activity of uroporphyrinogen by the protein encoded by At5g63290, although it has SAM cleavage activity (Fig. 2D). Lastly, Pratibha et al. claimed that At5g63290 encodes a mitochondrial protein, but when we transiently expressed the protein in *Nicotiana benthamiana*, the dominant signal appeared in the chloroplast (Supplementary Fig. S1 and Material and Methods).

**Figure 1.**
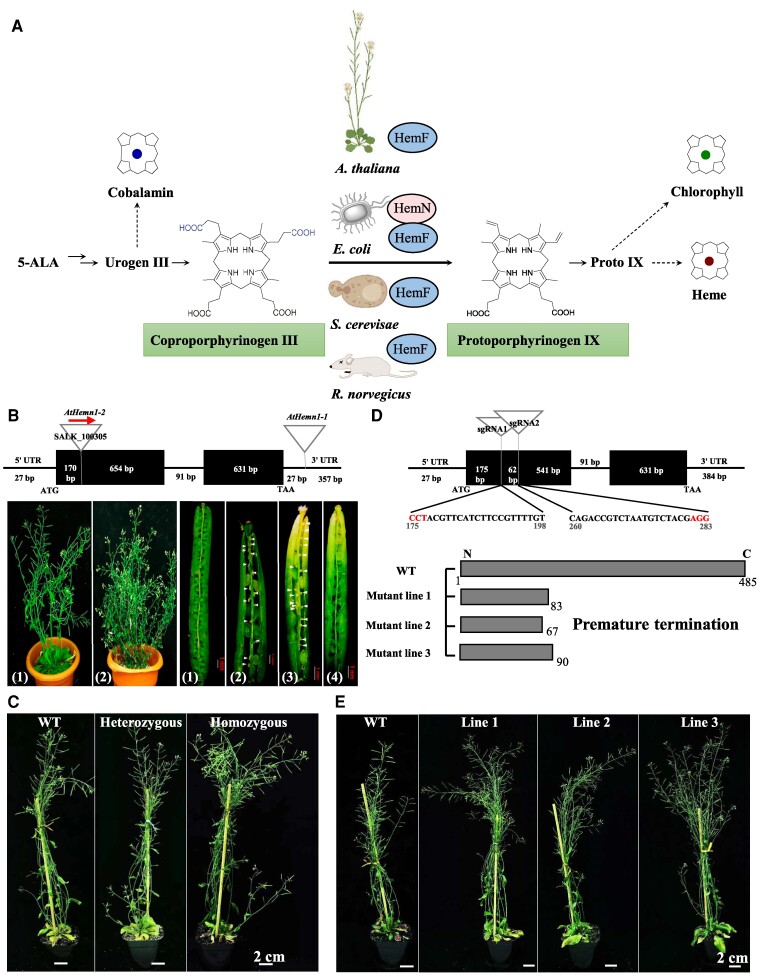
Genetic assessment of *At5g63290* for CPO activities that are crucial across life kingdoms. **A)** Schematic view of tetrapyrrole biosynthesis pathway with highlights on coproporphyrinogen III oxidase, which catalyzes the conversion of coproporphyrinogen III to protoporphyrinogen IX. HemF, oxygen-dependent CPO; HemN, oxygen-independent CPO; 5-ALA, 5-aminolevulinic acid; Urogen III, uroporphvrinogen III; Proto IX, protoporphyrin IX. **B)** Previously identified mutant lines (*Athemn1-1* and *Athemn1-2*) for *At5g63290*. Pictures were taken from Pratibha et al. It was reported that homozygotes were lethal, and only heterozygotes were phenotyped. (1), WT; (2), *Athemn1-1*; (3), *Athemn1-2*; (4), complementary of *Athemn1-1*. **C)** Phenotyping of *SALK_100305* in our lab. Both heterozygotes and homozygotes were viable. **D)** Characterization of 3 CRISPR/Cas9 introduced mutant lines, which all caused premature termination. **E)** Phenotyping of CRISPR/Cas9 KO mutant lines. No obvious growth and production phenotypes between wild type and mutants.

**Figure 2.**
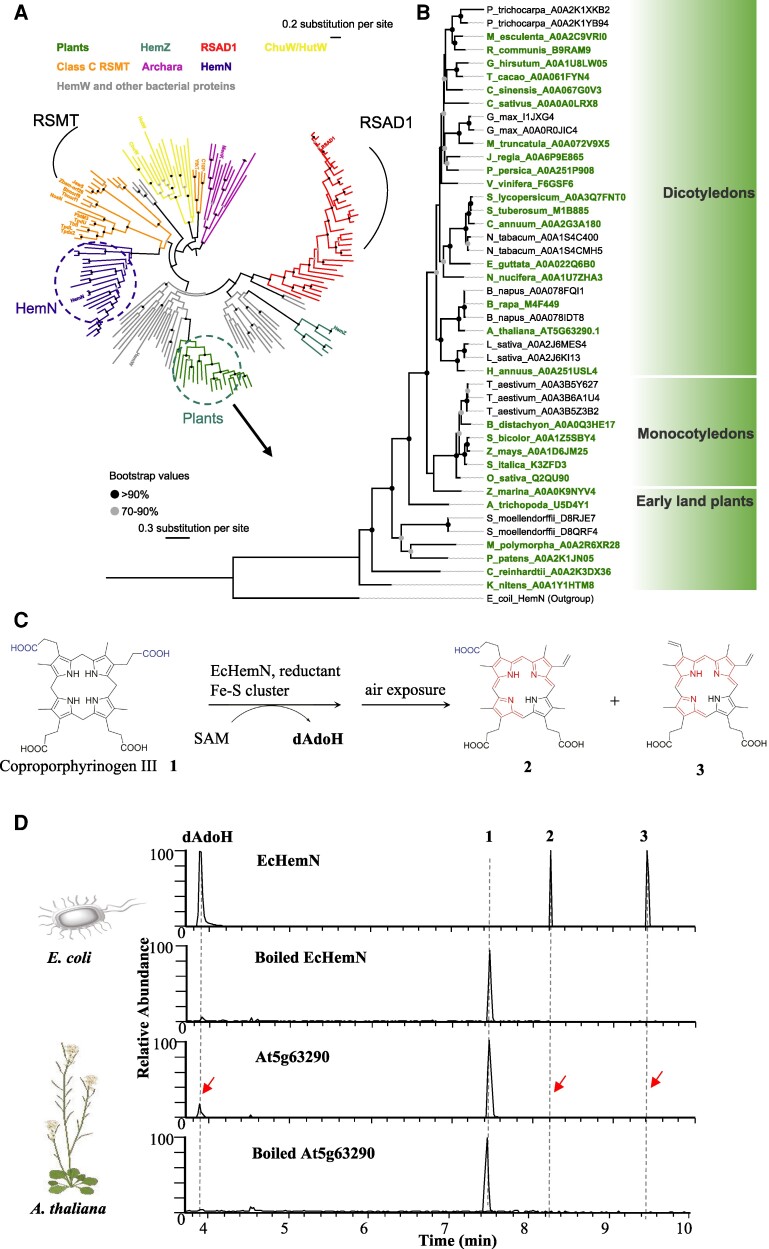
Phylogenetic and enzymatic analysis of At5g63290 for CPO activities. **A)** Phylogeny of Hem-like proteins across various life kingdoms. The sequences were taken from Cheng et al. The maximum likelihood method was used to construct the tree with 1,000 bootstrap replicates. **B)** Phylogeny of Hem-like proteins across land plants. Single-copy genes are highlighted in bold. **C)** Schematic view showing in vitro CPO reaction, with the mono- or didecarboxylated products (protoporphyrinogen IX) to be oxidized (indicated in red in compounds **2** and **3**) when exposed in air (James and Hift 2000). dAdoH was resulted from the SAM cleavage. **D)** Aligned chromatograms by analyzing reaction mixture from in vitro enzyme assays using LC-HRMS. The extracted ion chromatograms include [M + H]^+^ = 252.11 (corresponding to the dAdoH), [M + H]^+^ = 661.32 (corresponding to compound **1**), [M + H]^+^ = 609.27 (corresponding to compound **2**), and [M + H]^+^ = 563.27 (corresponding to compound **3**).

In view of the essential role of tetrapyrrole synthesis in plants, e.g. in respiration and photosynthesis, one would expect a severe growth phenotype of a mutant lacking a single gene for a specific step of tetrapyrrole biosynthesis (Tanaka and Tanaka 2007; Honkanen et al. 2016). Pratibha et al. (2017) presented the 2 homozygous T-DNA insertion mutants *Athemn1-1* (with a T-DNA insertion in the 3′ UTR of *At5g63290*) and *Athemn1-2* (with a T-DNA insertion in the first exon of *At5g63290*) as lethal mutants, while their heterozygous progenies contained shorter siliques, fewer ovules, and seed sterility under long-day conditions (Fig. 1B). We also obtained and conducted phenotyping of the same *Athemn1-2* T-DNA mutant (SALK_100305) (Supplementary Fig. S2, Material and Methods, and Table S1). In our experiments, both heterozygous and homozygous *Athemn1-2* mutants were fertile (Fig. 1C). To verify this analysis, we used CRISPR/Cas9-mediated mutagenesis and generated 3 independent knockout (KO) lines targeting the first exon of *At5g63290* (Fig. 1D). Also, none of these KO mutants were lethal, and both the heterozygotes and homozygotes showed no noticeable differences in growth or development compared to the wild-type control (Fig. 1E). Recent publications confirm that all plant tetrapyrrole biosynthesis is exclusively localized in plastids. Earlier findings suggesting a second heme synthesis pathway with the final enzymes protoporphyrinogen oxidase and ferrochelatase in mitochondria have been refuted (Hedtke et al. 2023; Wittmann et al. 2024). Therefore, there seems to be no need for mitochondrial CPO in plants.

Phylogeny and functional analysis further supported our findings. Cheng et al. (2022) conducted a Bayesian-based phylogenic analysis of HemN-like proteins across various life kingdoms. Their findings suggest that putative plant HemN-like proteins do not cluster with the bacterial HemN and are instead arranged separately from other functional clades, such as class C radical SAM methyltransferase (RSMT), with members mostly function as methyltransferases (Mathew et al. 2022). This result was also confirmed by our phylogenetic analysis with maximum probability. The At5g63290 was not grouped with HemN but instead clustered with the HemW clade in bacterial and the radical SAM domain-containing 1 (RSAD1) clade in animals (Fig. 2A). The RASD1 was suggested to have a heme chaperone activity similar to HemW (Haskamp et al. 2018). *At5g63290* exists as a broadly distributed single-copy gene across land plants (Fig. 2B). So far, functions of HemW are not well understood. It has been reported that the *E. coli* HemW shows no CPO activity either in vitro or in vivo (Haskamp et al. 2018). Emerging evidence suggests that HemW acts as a heme chaperone rather than as a biosynthetic protein (Haskamp et al. 2018). Motif analysis revealed that At5g63290 contains a HNXXYW domain, which is known to be involved in heme-binding activity of HemW (Supplementary Fig. S3) (Sharma et al. 2021).

The At5g63290 also contained a CxxxCxxC motif characteristic of the radical SAM superfamily. This motif utilizes 3 Cys residues to bind a [4Fe-4S] cluster, coordinating with SAM to reductively cleave its carbon–sulfur bond. This process generates a highly reactive 5′-deoxyadenosyl (dAdo) radical, which initiates a variety of reactions (Broderick et al. 2014; Nicolet 2020). When coexpressing the iron–sulfur cluster biogenesis pathway along with *At5g63290* in *E. coli*, the protein was found to be soluble (Supplementary Fig. S4 and Material and Methods). This suggests that the Fe–S cluster is necessary for proper protein folding.

To investigate the CPO activity and elusive role of the putative radical SAM enzyme, purified recombinant protein was incubated with the substrate coproporphyrinogen III in the presence of dithionite and SAM under anaerobic conditions. The mixture was then analyzed using ultraperformance liquid chromatography high-resolution MS (UPLC-HRMS). Both EcHemN and At5g63290 displayed SAM cleavage activity, as indicated by the presence of 5-deoxyadenosine (dAdoH) in the extracts, which is characteristic of the superfamily of radical SAMs (Fig. 2, C and D). However, the m/z 609.27, corresponding to the oxidized harderoporphyrinogen (**2**), and the m/z 563.26, corresponding to the oxidized protoporphyrinogen IX (**3**), were only detected in EcHemN assay, not with At5g63290. Compounds **2** and **3** were the oxidized forms of HemN products, resulting from exposure to air before UPLC-HRMS analysis (Fig. 2, C and D).

Thus, based on our data, we conclude that *At5g63290* does not encode a CPO. But the biochemical function of this single-copy gene remains to be characterized further. We do not exclude that exposure of the T-DNA and CRISPR KO lines to severe stress conditions may exhibit visible mutant phenotypes, which could provide insights into the biofunctions of this gene.

## Supplementary Material

kiaf046_Supplementary_Data

## Data Availability

All data are incorporated into the article and its online Supplementary material.

## Supplementary data

The following materials are available in the online version of this article.

**Supplementary Material and Methods.**

**Supplementary Figure S1.** At5g63290 was targeted to both mitochondria and chloroplast.

**Supplementary Figure S2.** Identification of T-DNA insertion mutant plants by PCR analysis.

**Supplementary Figure S3.** Motif analysis of At5g63290.

**Supplementary Figure S4.** SDS-PAGE of At5g63290 protein expressed in *E. coli* Rosetta (DE3) cells.

**Supplementary Table S1.** Primers used for vector construction and genotyping.

## Dive Curated Terms

The following phenotypic, genotypic, and functional terms are of significance to the work described in this paper:

At5g63290 Gramene: AT5G63290
At5g63290 Araport: AT5G63290

S-adenosyl-l-methionine CHEBI: CHEBI:15414
