# Supplementary material for: At5g63290 does not encode coproporphyrinogen III oxidase
Source: Plant Physiol. 2025 Jan 29;197(2):kiaf046. doi: 10.1093/plphys/kiaf046 (PMC11834973; doi:10.1093/plphys/kiaf046)
Supplement: kiaf046_Supplementary_Data [file kiaf046_supplementary_data.pdf]

Supplementary Information for

At5g63290 does not encode coproporphyrinogen III oxidase

Wenjuan Ji[1,#], Huijuan Wang[1,#], An Ning[1], Xuan Zhou[1], Bingxiao Wen[2], Bernhard Grimm[2] & Zhenhua Liu[1*]

1 Joint Center for Single Cell Biology; Shanghai Collaborative Innovation Center of Agri-Seeds, School of Agriculture and Biology, Shanghai Jiao Tong University, Shanghai 200240, China
2 Institute of Biology/Plant Physiology, Humboldt University Berlin, Berlin, Germany
#These authors contributed equally to this work
* Author for Correspondence: zhenhua.liu@sjtu.edu.cn

Material and Methods

Plant materials and growth conditions

The *Arabidopsis* T-DNA insertion mutant (SALK_100305) seeds were purchased from the *Arabidopsis* Biological Resource Center. The *At5g63290* knockout mutants in the Col-0 background were generated by CRISPR/Cas9 mutagenesis. All plants were grown in soil and cultivated in a greenhouse at 22°C, with 16 h of light/8 h of dark.

Gene Cloning

Total RNA was extracted from *Arabidopsis* leaves using the FastPure Universal Plant RNA Kit (Vazyme, Nanjing, China). Reverse transcription reactions were performed using the HiScript III RT SuperMix Kit for qPCR (Vazyme, Nanjing, China) to synthesize cDNA following the manufacturer's protocol. Full-length ORFs of *At5g63290* were amplified using High-Fidelity DNA polymerase (Vazyme, Nanjing, China), and the primers were listed in Supplementary Table 1.

CRISPR/Cas9 genome-editing vector construction and plant transformation

To generate the *At5g63290* knockout mutants, the targetDesign website (http://skl.scau.edu.cn/targetdesign/) was used to design sgRNAs. The Cas9 expression cassettes, the sgRNA expression cassettes and a FAST-Red selectable maker were assembled into pICSL4723 binary vector according to the Golden Gate modular cloning method (Engler et al., 2014; Castel et al., 2019). The CRISPR/Cas9 construct was transformed into *Arabidopsis* Col-0 plants by *Agrobacterium tumefaciens* GV3101 using floral dip method (Zhang et al., 2006). The red seeds (due to the Fast-red gene) were selected from T0 seeds for subsequent analysis. Non-red seeds from the T1 seeds were selected for targeted gene sequencing. The resulting T2 plants were checked and homozygous mutant plants without foreign vector sequences were used for phenotyping. The mutants editing sites were confirmed by PCR and sequencing analysis.

Characterization of the T-DNA insertion mutant

The T-DNA insertion site for SALK_100305 was identified by PCR. Genomic DNA was obtained from the mutants using the extraction buffer (200 mM Tris-HCl, 250 mM NaCl, 25 mM EDTA, 0.5% SDS, pH 8.0) and used as a template. The PCR reaction was performed using the 2 × Rapid Taq Master Mix (Vazyme, Nanjing, China) with the primers (listed in Supplementary Table 1).

Cellular Localization of At5g63290

At5g63290 without the stop codon was amplified from leaf cDNA by Phanta High-Fidelity DNA polymerase kit (Vazyme, Nanjing, China) and cloned into the pEAQ-XhoI vector to construct the C-terminal mNeonGreen fusion protein under the control of the constitutive promoter CaMV35S. *AtRIP1* (NM_112362.4), located in the mitochondria, were selected as organelle marker (Nelson et al., 2007). The constructed vectors and mitochondria marker were transferred to *Agrobacterium tumefaciens* GV3101, respectively. *A. tumefaciens* strains were grown in LB medium containing 50 μg/mL kanamycin and 50 μg/mL rifampicin at 28°C until the OD_{600} reached 0.8-1.0. The cells were harvested at 4,000g for 10 min and resuspended in infiltration buffer (10 mM $MgCl_2$, 10 mM MES, and 200 μM acetosyringone) and incubated at room temperature for an additional 2 hours. The strains were infiltrated into the leaves of six-week-old *Nicotiana benthamiana* by a syringe without needle. The plants were cultivated in a greenhouse in the dark for two days. Fluorescence was observed with a laser confocal scanning microscope (LSM800, Zeiss, Germany) with excitation/emission at 488/507 nm for mNeongreen, 587/610 nm for mCherry, and 640/675 nm for chloroplast, respectively

Phylogenetic analysis of At5g63290

MUSCLE program was used to align the full-length protein sequences with default parameters (Edgar, 2004). Phylogenetic trees were constructed using the maximum likelihood method and Jones-Taylor-Thornton model using FastTree (Hua et al., 2022). A bootstrap resampling analysis with 1000 replicates was performed to evaluate the topology of phylogeny. The online tool tvBOT (https://www.chiplot.online/tvbot.html) was used to display the phylogenetic tree (Xie et al., 2023). Plant Orthologous genes of *At5g63290* were identified by TAIR (https://www.arabidopsis.org/) and downloaded from Uniprot (https://www.uniprot.org/).

Expression of At5g63290

The plasmid pDB1282 contains the isc operon from *A. vinelandii* under control of an arabinose-inducible promoter with an ampicillin resistance cassette. It was used to assist the assembly of iron-sulfur clusters in heterologously expressed SAM proteins (Delli-Bovi et al., 2010). *E. coli* Rosetta (DE3) cells were used for transformation. A single colony transformant with both pET28a-At5g63290 and pDB1282 plasmids was used to inoculate a 5 mL LB culture supplemented with 50 μg/mL kanamycin. The culture was grown at 37°C for 12 hours and was used to inoculate 500 mL of LB medium containing 50 μg/mL kanamycin. Cells were grown at 37°C and 180 rpm to an OD_{600} 0.6-0.8. IPTG and $Fe(NH_4)_2(SO_4)_2$ solution was added at a final concentration of 0.2 mM. After additional 20-24 hours of incubation at 20°C at 130 rpm, the cells were harvested by centrifugation at 4,500 rpm for 10 min at 4°C. The cell pellet was resuspended in 15 mL of the lysis buffer (50 mM Tris-HCl, 200 mM NaCl, and 10% glycerol,

pH 8.0), and was lysed by sonication on ice. The lysis suspension was centrifugated at 14,000 rpm for 30 min at 4°C. The supernatant and precipitate were analyzed by SDS-PAGE (12%Tris-glycine gel) analysis (Figure S4). The EcHemN was expressed and analyzed as a positive control (Ji et al., 2020).

Purification and reconstitution of At5g63290

Purification of At5g63290 and reconstitution of iron-sulfur cluster in protein were performed in an anaerobic glove box (Coy Laboratory Product Inc., USA) with less than 2 ppm of O_2. The cell pellet was resuspended in 15 mL of the lysis buffer (50 mM Tris-HCl, 200 mM NaCl, and 10% glycerol, pH 8.0), and was lysed by sonication on ice. Cell debris was removed via centrifugation at 14,000 rpm for 30 min at 4°C. The supernatant was incubated with 4 mL Ni-NTA resin pre-equilibrated with the lysis buffer, and then subjected to affinity purification on this column. The desired fractions were combined and protein concentration was determined using a Bradford Assay Kit (Bio Rad) using bovine serum albumin (BSA) as a standard.

As centrifugation outside the glove box which may contaminate the lysis buffer with O_2, thereby destroying the iron-sulfur cluster from the above-purified protein (Imlay, 2006), we provided additional Fe-S source to reconstitute functional proteins following previously described protocol (Ji et al., 2020). Briefly, freshly prepared dithiothreitol (DTT) was added to the purified protein fraction at a final concentration of 5 mM. $Fe(NH_4)_2(SO_4)_2$ solution (50 mM) was added carefully to reach a final concentration of 500 μM. After 10 min of incubation on ice, Na_2S solution (50 mM) was added carefully to reach a final concentration of 500 μM. After further incubation on ice for 3-5 hours, the resulting blackish solution was subjected to desalting on a PD-10 column (GE Healthcare), which was pre-equilibrated with the desalting buffer (50 mM Tris, 25 mM NaCl, 10 mM DTT and 10% (v/v) glycerol, pH 8.0). The protein fraction was collected, analyzed by SDS-PAGE (12%Tris-glycine gel) (Figure S4) and was used directly for *in vitro* assay or stored at -80 °C upon further use.

Enzyme assays and LC-MS analysis

Coproporphyrinogen III was prepared by reduction of coproporphyrin III by using sodium borohydride according to the method reported previously (Ji et al., 2019). All assays were performed in a Coy anaerobic chamber with less than 2 ppm of O_2. Enzyme assays were conducted in 100 μL reaction mixture containing 50 μg desalted protein, 20 μM coproporphyrinogen III, 1mM SAM and 4 mM sodium dithionate in reaction buffer (50 mM Tris, 25 mM NaCl, 10 mM DTT and 10% (v/v) glycerol, pH 8.0). Reactions were initiated by adding SAM and incubated at room temperature for overnight prior to quenching by addition of trifluoroacetic acid (TFA) at a final concentration of 5% (v/v). After removal of the protein precipitates by centrifugation, the supernatant was subjected to LC-HR-MS analysis. The supernatant was exposed to air for 24 hours, and the oxidized products (i.e. porphyrins) were prepared for the LC-HR-MS analysis.

The UPLC-HR-MS analyses were performed by a Thermo Fisher UltiMate 3000 UPLC system coupled to a Q-Exactive Plus Hybrid Quadrupole Orbitrap Mass Spectrometer (Thermo Fisher). A UPLC BEH C18 column (2.1 mm i.d. × 100 mm, 1.7 μm, Waters) with a VanGuard BEH

C18 precolumn (2.1 mm i.d. × 5 mm, 1.7 μm, Waters) was used for separation. The mobile phase consisted of 0.1% formic acid in water (phase A) and acetonitrile (phase B). The column temperature was kept at 30°C and the flow rate was 0.4 mL/min. The chromatographic gradient was showed as follows: 2% B for 2 min, 2% B to 40% B for 4 min, changing to 95% B for 1 min, hold 95% B for 3 min, finally returning to the initial conditions (2% B) for 2 min. Products were detected using electrospray ionization in positive mode (ESI^+) with the ion spray voltage set at 3,500 V, and the ions were fragmented by collision induced dissociation (CID) (normalized collision energy: 17.5, 35, 52.5, isolation window: 3 m/z). The MS scan range was from 100 to 1500.

Supplementary Figures

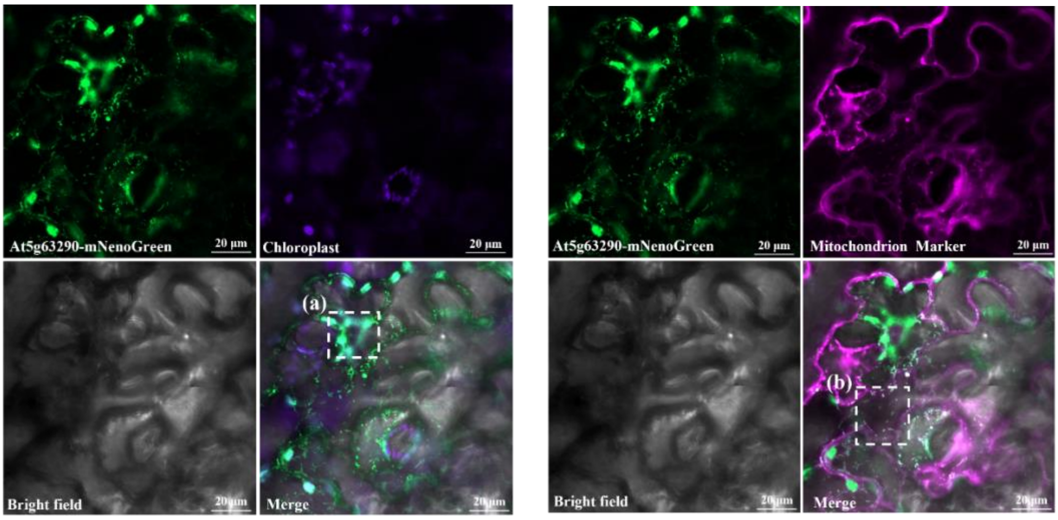

Supplementary Figure S1 At5g63290 was targeted to both mitochondria and chloroplast. Subcellular localization of At5g63290 as determined by transient expression of mNeonGreen-tagged proteins in *N. benthamiana* leaves. *AtRIP* was used as the mitochondria organelle marker, and a chlorophyll fluorescence as a marker for chloroplasts. Typical chloroplast (a) and mitochondria (b) signals are indicated by dashed frames.

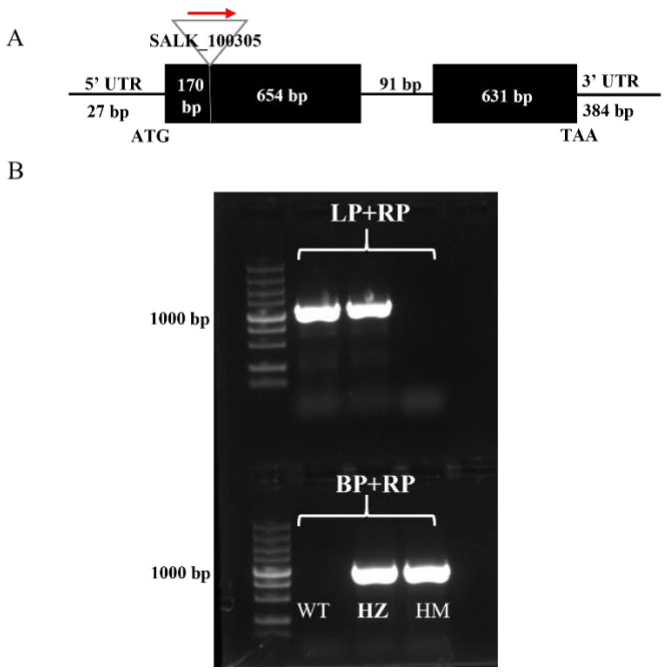

Supplementary Figure S2 Identification of T-DNA insertion mutant plants by PCR analysis.

A, Schematic representation of the T-DNA insertion site in SALK_100305 mutants. **B,** PCR with the primers LP and RP for wild-type band, and primers BP and RP for mutant band.

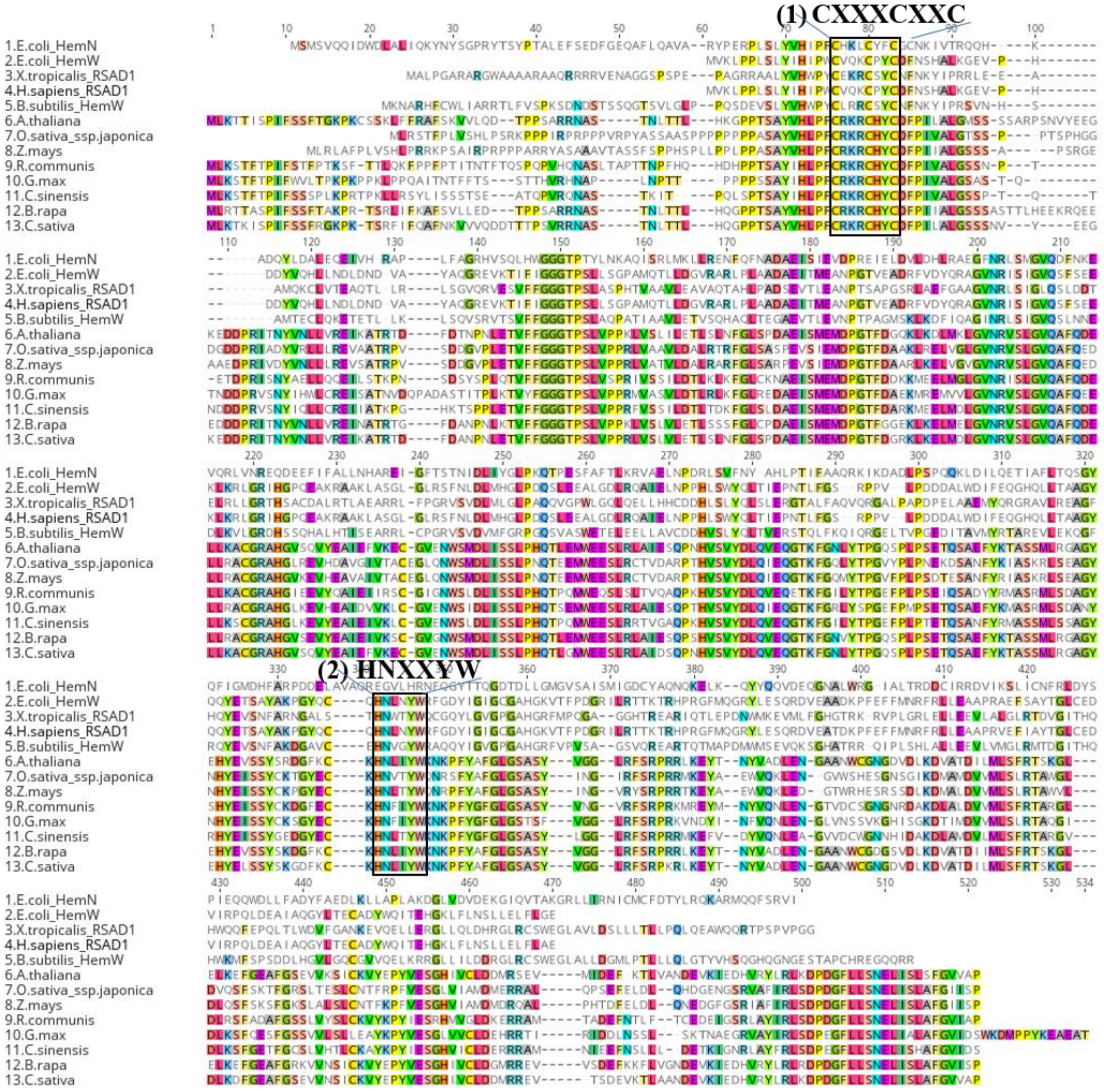

Supplementary Figure S3 Motif analysis of At5g63290.

Geneious Prime (version: 2024.0.5) (Fister et al., 2018) was used for protein sequence alignment. Hem-like proteins from *E. coli*, *Xenopus tropicalis*, *Homo sapiens* and some plant species (*Arabidopsis thaliana, Oryza sativa, Zea mays, Ricinus communis, Glycine max, Camellia sinensis, Brassica rapa, Camellia sativa*) were used. The CXXXCXXC and HNXXYW motifs are accepted as heme-binding domains and labeled by (1) and (2), respectively.

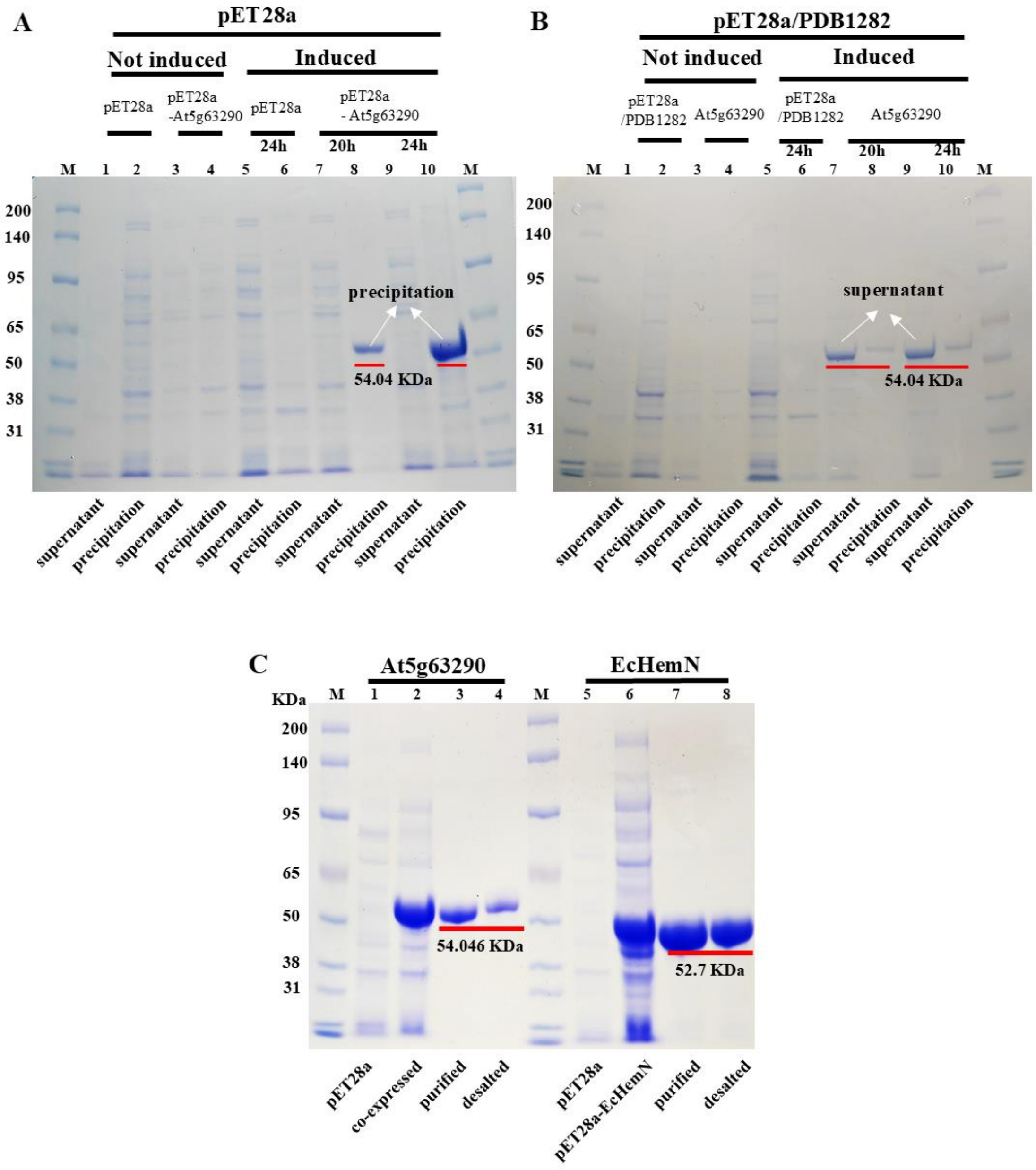

Supplementary Figure S4 SDS-PAGE of At5g63290 protein expressed in *E. coli* Rosetta (DE3) cells.

A, Without co-expression of the pDB1282 plasmid, At5g63290 protein was mainly detected in the pellet. **B,** By co-expressing of the pDB1282 plasmid, At5g63290 protein was mainly detected in supernatant. **C**, purified At5g63290 protein and the desalted At5g63290 protein, EcHemN as the positive control.

Supplementary Table 1

Primers used for vector construction and genotyping.

Primers	Sequence	Description
sgRNA1	TGTGGTCTCAATTGCAGACCGTCTAATGTC TACGGTTTAAGAGCTATGCTGGAA	CRISPR/Cas9 genome-editing vector construction
sgRNA2	TGTGGTCTCAATTGACAAAACGGAAGATG AACGTGTTTAAGAGCTATGCTGGAA	
L2R	GGATAAACCTTTTCACGCCC	Identification for CRISPR/Cas9 mutant
Cas9-F	CTCATCGTTTACAGGTAAACCC	
Cas9-R	AACTCAATGTTTCCAGAATC	
pET28a-At5g63290-F	GGTCGCGGATCCGAATTCATGCATCATCAT CATCATCACCTGAAAACAACGATTTCTC	Cloning At5g63290 for heterologous expression
pET28a-At5g63290-R	TCGAGTGCGGCCGCAAGCTTTTAAGGAGC TACCACGCCAAACGATAGAGA	
pEAQ-XhoI-mNenoGreen-At5g63290-F	CAAATTCGCGCTCGAGGTCGACATGCTGA AAACAACGATTTCTCCA	Subcellular localization in tobacco
pEAQ-XhoI-mNenoGreen-At5g63290-R	ACCTTTGCTGACCATGTCGACAGGAGCTA CCACGCCAAACGATA	
pET28a-EcHemN-F	GTGCCGCGCGGCAGCCATATGATGTCTGT ACAGCAAATCGACTG	EcHemN Cloning
pET28a-EcHemN-R	GTGGTGGTGGTGGTGCTCGAGTTAAATCA CCCGAGAGAACTGC	
BP	ATTTTGCCGATTTCGGAAC	T-DNA mutant identification
LP	GCTCTGAAACACGACCTCTTG	
RP	TGAGCTCTTCCACAAGCTTTC	

References

Castel B, Tomlinson L, Locci F, Yang Y, Jones JDG (2019) Optimization of T-DNA architecture for Cas9-mediated mutagenesis in Arabidopsis. PLoS One **14**: 1–20

Delli-Bovi TA, Spalding MD, Prigge ST (2010) Overexpression of biotin synthase and biotin ligase is required for efficient generation of sulfur-35 labeled biotin in E. coli. BMC Biotechnol **10**: 73

Edgar RC (2004) MUSCLE: Multiple sequence alignment with high accuracy and high throughput. Nucleic Acids Res **32**: 1792–1797

Engler C, Youles M, Gruetzner R, Ehnert TM, Werner S, Jones JDG, Patron NJ, Marillonnet S (2014) A Golden Gate modular cloning toolbox for plants. ACS Synth Biol **3**: 839–843

Fister AS, Landherr L, Maximova SN, Guiltinan MJ (2018) Transient expression of CRISPR/Cas9 machinery targeting TcNPR3 enhances defense response in Theobroma cacao. Front Plant Sci **9**: 268

Hua X, Song W, Wang K, Yin X, Hao C, Duan B, Xu Z, Su T, Xue Z (2022) Effective prediction of biosynthetic pathway genes involved in bioactive polyphyllins in Paris polyphylla. Commun Biol **5**: 1–10

Imlay JA (2006) Iron-sulphur clusters and the problem with oxygen. Mol Microbiol **59**: 1073–1082

Ji W, Ji X, Zhang Q, Mandalapu D, Deng Z, Ding W, Sun P, Zhang Q (2020) Sulfonium-based homolytic substitution observed for the radical SAM enzyme HemN. Angew Chemie - Int Ed **59**: 8880–8884

Ji X, Mo T, Liu WQ, Ding W, Deng Z, Zhang Q (2019) Revisiting the mechanism of the anaerobic coproporphyrinogen III oxidase HemN. Angew Chemie - Int Ed **58**: 6235–6238

Nelson BK, Cai X, Nebenführ A (2007) A multicolored set of in vivo organelle markers for co-localization studies in Arabidopsis and other plants. Plant J **51**: 1126–1136

Xie J, Chen Y, Cai G, Cai R, Hu Z, Wang H (2023) Tree Visualization By One Table (tvBOT): a web application for visualizing, modifying and annotating phylogenetic trees. Nucleic Acids Res **51**: W587–W592

Zhang X, Henriques R, Lin SS, Niu QW, Chua NH (2006) Agrobacterium-mediated transformation of Arabidopsis thaliana using the floral dip method. Nat Protoc **1**: 641–646
